# OsMKK6 Regulates Disease Resistance in Rice

**DOI:** 10.3390/ijms241612678

**Published:** 2023-08-11

**Authors:** Ruirui Jiang, Shichen Zhou, Xiaowen Da, Peng Yan, Kai Wang, Jiming Xu, Xiaorong Mo

**Affiliations:** State Key Laboratory of Plant Physiology and Biochemistry, College of Life Science, Zhejiang University, Hangzhou 310058, China; jiangrr@zju.edu.cn (R.J.); 3160102592@zju.edu.cn (S.Z.); dxwpp@zju.edu.cn (X.D.); yanpengzju@zju.edu.cn (P.Y.); wangkaiwyh@zju.edu.cn (K.W.); xujiming@zju.edu.cn (J.X.)

**Keywords:** rice (*Oryza sativa* L.), lesion mimic mutant, defense response, OsMKK6

## Abstract

Mitogen-activated protein kinase cascades play important roles in various biological programs in plants, including immune responses, but the underlying mechanisms remain elusive. Here, we identified the lesion mimic mutant *rsr25* (*rust spots rice 25*) and determined that the mutant harbored a loss-of-function allele for *OsMKK6 (MITOGEN-ACTIVATED KINASE KINASE 6)*. *rsr25* developed reddish-brown spots on its leaves at the heading stage, as well as on husks. Compared to the wild type, the *rsr25* mutant exhibited enhanced resistance to the fungal pathogen *Magnaporthe oryzae* (*M. oryzae*) and to the bacterial pathogen *Xanthomonas oryzae* pv. *oryzae* (*Xoo*). OsMKK6 interacted with OsMPK4 (MITOGEN-ACTIVATED KINASE 4) in vivo, and OsMKK6 phosphorylated OsMPK4 in vitro. The *Osmpk4* mutant is also a lesion mimic mutant, with reddish-brown spots on its leaves and husks. Pathogen-related genes were significantly upregulated in *Osmpk4*, and this mutant exhibited enhanced resistance to *M. oryzae* compared to the wild type. Our results indicate that OsMKK6 and OsMPK4 form a cascade that regulates immune responses in rice.

## 1. Introduction

Plants lack the ability to flee from pathogen attack and have, therefore, evolved a two-layered immune system to defend themselves against pathogens. The first layer of their immune system is known as pathogen-associated molecular pattern-triggered immunity (PAMP-triggered immunity, PTI). Pattern recognition receptors (PRRs) located on the cell membrane recognize pathogen-associated molecular patterns (PAMPs) and activate a series of immune responses [1]. In turn, pathogens have evolved a class of effectors, which are secreted by pathogens into plant cells and inhibit the plant PTI immune response by attacking and suppressing the PTI signaling pathway. In response, R (Resistance) proteins in plants directly or indirectly monitor effectors and trigger immune responses called effector-triggered immunity (ETI) [2]. ETI is more rapid and induces a stronger immune response than PTI, with cell death often occurring at the site of infection, a phenomenon known as the hypersensitive response (HR) [3]. The HR is an effective way for plants to defend themselves against pathogens and is accompanied by programmed cell death (PCD), which limits the spread of pathogens to non-infected tissues [4].

Lesion mimic mutants (LMMs) spontaneously produce HR-like lesions accompanied by PCD [5]. Therefore, LMMs are ideal materials to elucidate the molecular mechanism of PCD and defense responses in plants. Many LMMs have been identified in various plant species, such as Arabidopsis (*Arabidopsis thaliana*) [6,7], maize (*Zea mays*) [8], wheat (*Triticum aestivum*) [9], barley (*Hordeum vulgare*) [10], and rice (*Oryza sativa*) [11,12,13]. Rice is a primary calorie provider for approximately one-half of people across the world [14]. Different fungal, bacterial, and viral pathogens cause large reductions in rice grain production. *Magnaporthe oryzae* (*M. oryzae*), a filamentous ascomycete fungus, is the causal agent of rice blast disease, the most destructive disease of rice, which can cause a 10–30% reduction in rice yields [15] *Xanthomonas oryzae* pv. *oryzae* (*Xoo*) is a bacterial disease that causes a 10–50% reduction in rice yields [16]. Therefore, exploring the molecular mechanisms of immune response in rice is crucial for improving rice yield. Several LMM genes have been cloned in rice. These genes encode proteins that perform diverse functions, highlighting the complex molecular mechanisms of PCD and defense responses. For example, Cullin3-based RING E3 ubiquitin ligases (CRL3s), consisting of Cullin3 (CUL3), RBX1, and BTB domain-containing proteins, are involved in plant immunity. In rice, OsCUL3a interacts with proteins such as RING-BOX 1a (RBX1a) and RBX1b to form a CRL, which negatively regulates cell death and immune responses by degrading NONEXPRESSER OF PR GENES 1 (OsNPR1) [17]. Disrupting *EARLY LESION LEAF 1* (*OsELL1*), encoding a cytochrome P450 monooxygenase [15], induces reactive oxygen species (ROS) accumulation and cell death [18]. *SPOTTED LEAF 11* (*SPL11*) encodes an E3 ubiquitin ligase that negatively regulates PCD and innate immunity [19]. SPL11 ubiquitinates the S-domain receptor-like kinase SDS2 (SPL11 CELL-DEATH SUPPRESSOR 2), leading to its degradation via the 26S proteasome. In addition, SDS2 phosphorylates and activates the related RECEPTOR-LIKE CYTOPLASMIC KINASE 118 (OsRLCK118) and OsRLCK176, which positively regulate immunity by stimulating ROS production via phosphorylation of the NADPH oxidase RESPIRATORY BURST OXIDASE HOMOLOG B (OsRBOHB) [20]. *SPL3* encodes a mitogen-activated protein kinase kinase kinase that negatively regulates plant resistance to pathogens by suppressing the activation of the OsMPKK10.2–OsMPK6 cascade [21].

Mitogen-activated protein kinase (MAPK) cascades play key roles in various physiological processes in plants, such as regulating plant growth and development, biotic and abiotic stress responses, and phytohormone signaling [22,23]. MAPK cascades consist of three classes of conserved kinases: MAP kinase kinase kinase (MAPKKK or MKKK), MAP kinase kinase (MAPKK or MKK), and MAP kinase (MAPK or MPK). MKKK phosphorylates MKK, and the activated MKK phosphorylates and activates MPK. MPK usually interacts with specific downstream components such as transcription factors for signal transduction [24,25].

Several MAPK cascades regulate immune responses in plants. For example, in Arabidopsis, the MEKK1–MKK1/MKK2–MPK4 cascade negatively regulates plant innate immunity [26,27]. The components of this cascade were identified based on the autoimmune phenotypes of the *mekk1*, *mkk1mkk2*, and *mpk4* mutants [26,27,28,29]. MKK6 functions with MEKK1 and MPK4 to prevent autoimmunity, and the ANP2 (ARABIDOPSIS NUCLEUS- AND PHRAGMOPLAST-LOCALIZED KINASE1-RELATED PROTEIN KINASE 2)/ANP3–MKK6–MPK4 cascade is critical for regulating plant immunity [30].

The protein homologous to Arabidopsis MPK4 in rice is OsMPK4 (encoded by Os10g0533600) [31]. OsMPK4 kinase activity in *Osmpk4* is reduced due to an R89K mutation. The *Osmpk4* was isolated from screening for lesion mimic mutants and it exhibited enhanced resistance to *Xoo*, whereas *OsMPK4*-overexpressing transgenic plants were more susceptible to *Xoo* than wild type plants, suggesting that OsMPK4 negatively regulates resistance to *Xoo* in rice [32]. In addition, OsMPK4 is a target protein of the virulence factor SCRE6 (small cysteine-rich effector 6) from *Ustilaginoidea virens*. SCRE6 dephosphorylates OsMPK4 to enhance its stability, thus suppressing immunity [33]. Moreover, the OsMKK6–OsMPK4–OsVQ14 (VALINE-GLUTAMINE [VQ] MOTIF-CONTAINING PROTEIN 14)/OsVQ32 cascade positively regulates the resistance of rice to *Xoo* [34]. However, the immune responses of loss-of-function mutants of *OsMKK6* are unclear, and determining whether the OsMKK6–OsMPK4 cascade is involved in immune responses to the fungal pathogen *M. oryzae* requires further study. 

To further understand the mechanism of immune responses in rice, we cloned the LMM gene *OsMKK6* following the isolation of the *rsr25* (*rust spots rice 25*) mutant. The mutation of *OsMKK6* in *rsr25* resulted in enhanced resistance to *M. oryzae* and *Xoo*, with a stronger pathogen-induced defense response in *Osmkk6* than in the wild type. OsMKK6 interacted with OsMPK4 in vivo, and OsMKK6 phosphorylated OsMPK4 in vitro. The D69N mutation in OsMKK6 in the *rsr25* mutant prevented this protein from interacting with OsMPK4. The *Osmpk4* mutant exhibited an LMM phenotype and enhanced resistance to *M. oryzae* compared to wild type plants. Therefore, we identified the phenotype of a loss-of-function mutant of *OsMKK6* and demonstrated that the OsMKK6–OsMPK4 cascade is involved in regulating the resistance of rice to the fungal pathogen *M. oryzae*. Our finding that the OsMKK6–OsMPK4 cascade regulates resistance to both fungal and bacterial pathogens underscores its importance in broad-spectrum disease resistance.

## 2. Results

### 2.1. Phenotypic Characterization of the rsr25 Mutant

**Figure 1:**
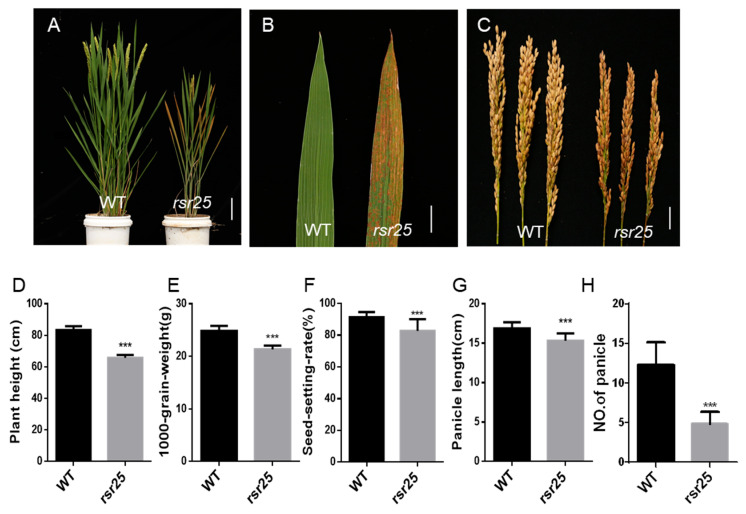
Phenotypic characterization of the *rsr25* mutant. (**A**) Wild type (WT) and *rsr25* mutant plants at mature stage. Scale bar = 10 cm. (**B**) Leaves of WT and *rsr25* mutant plants at mature stage. Scale bar = 2 cm. (**C**) WT and *rsr25* mutant plant panicles at mature stage. Scale bar = 2 cm. (**D**–**H**) Comparisons of plant height (**D**), 1000-grain weight (**E**), seed-setting rate (**F**), panicle length (**G**), and the number of panicles (**H**) of WT and *rsr25* mutant plants. Data represent means ± SD (*n* = 15 in (**D**,**F**–**H**); *n* = 5 in (**E**)), *** *p* < 0.001, Student’s *t*-test.

We identified the *rsr25* mutant from an EMS mutant library in the Xiushui134 (XS134) background. Compared to the wild type (WT), *rsr25* leaves developed reddish-brown spots at the heading stage in the absence of pathogen attack. The spots spread from the leaf tip to the leaf base and from old leaves to new leaves until they were distributed throughout the plant at the mature stage (Figure 1A,B). Reddish-brown spots also appeared on the husks of the *rsr25* mutant (Figure 1C). We measured the agronomic traits of WT and *rsr25* plants grown in soil, finding that *rsr25* had a lower plant height and seed-setting rate, lower 1000-grain weight, shorter panicles, and fewer panicles than WT plants (Figure 1D–H). These results suggest that the *rsr25* mutant exhibits an LMM phenotype and that the formation of the lesion spots seriously affects the growth and development of *rsr25* compared to the WT. 

### 2.2. OsRSR25 Encodes OsMKK6, a MAP Kinase Kinase

**Figure 2:**
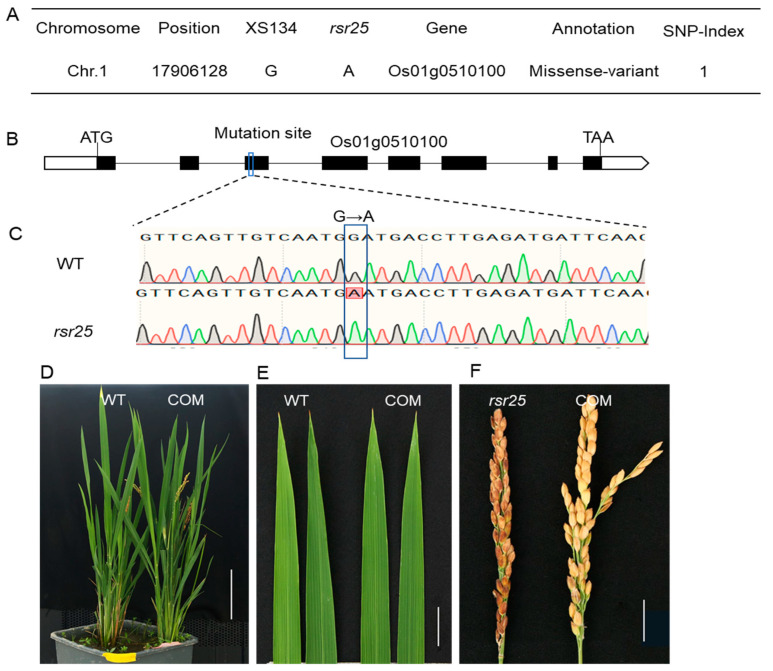
Cloning of *OsRSR25* gene. (**A**) Identification of the mutation site in *rsr25* mutant using the MutMap approach. Whole-genome sequencing detected a missense mutation (G to A) in gene *Os01g0510100*, and the SNP index (the proportion of SNPs in the mutant population) was 1. (**B**) Structure of the *Os01g0510100* gene and the mutation site, white boxes represent 5′-UTR (left) and 3′-UTR (right); black boxes represent exons; black lines represent introns. (**C**) Sequence comparison of the WT and *rsr25* mutant in the mutation site. (**D**) The phenotype of WT and COM T_1_ generation transgenic complementation plants, scale bar = 10 cm. (**E**) Leaves of WT and COM T_1_ generation transgenic complementation plants, scale bar = 2 cm. (**F**) Panicles of *rsr25* mutant and COM T_1_ generation transgenic complementation plants, scale bar = 2 cm.

To identify the gene responsible for the phenotypes of the *rsr25* mutant, we crossed *rsr25* with XS134 plants to generate a segregating F_2_ population for MutMap sequencing. The F_1_ progeny exhibited the same phenotype as the WT XS134, and the segregation ratio of normal to lesion mimic individuals in the F_2_ population was approximately 3:1 (Table S1), suggesting that a single recessive nuclear gene is responsible for the *rsr25* phenotype. MutMap sequencing of F_2_ individuals with WT and mutant phenotypes revealed a missense mutation (G to A, 205 bp downstream from the start codon of the coding sequence) in the third exon of *OsMKK6* (Os01g0510100) (Figure 2A–C), which caused an aspartate (Asp, D) to asparagine (Asn, N) change at amino acid residue 69 of OsMKK6 in *rsr25* (Figure S1). 

To further test whether the mutation in *OsMKK6* is responsible for the *rsr25* mutant phenotype, we constructed the complementation (COM) plasmid *proOsMKK6:OsMKK6CDS-GFP*, which includes the full-length coding sequence of *OsMKK6* driven by its native promoter. We obtained 15 COM transgenic lines in the *rsr25* mutant background. The phenotype of all COM plants was identical to that of WT plants, with no lesion spot formation on their leaves or husks (Figure 2D–F). These results demonstrate that the mutation in *OsMKK6* is responsible for the *rsr25* phenotype.

### 2.3. OsMKK6 Regulates Immunity against Magnaporthe oryzae and Xanthomonas oryzae pv. oryzae

**Figure 3:**
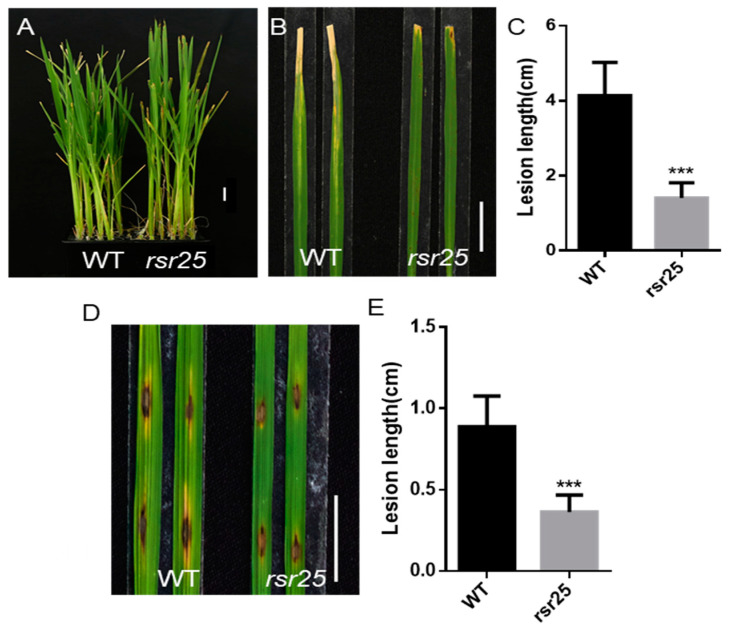
The *rsr25* mutant displays enhanced resistance to both *M. oryzae* and *Xoo*. (**A**–**C**) Leaves of *rsr25* mutant plants and WT plants at 30 dps (days post-sowing) were inoculated with *Xoo* isolate P6. (**A**) The whole plants were photographed 14 days post-inoculation (dpi). Scale bar = 2 cm. (**B**) Leaves were photographed at 14 dpi. Scale bar = 2 cm. (**C**) Lesion length on the leaves of WT and *rsr25* mutant plants at 14 dpi shown in (**A**,**B**). Data represent means ± SD (*n* = 13), *** *p* < 0.001, Student’s *t*-test. (**D**,**E**) Punch inoculation of WT and *rsr25* mutant plants at 30 dps with the *M. oryzae* isolate RB22. (**D**) Leaves were photographed at 7 dpi. Scale bar = 2 cm. (**E**) Lesion length on the leaves of WT and *rsr25* mutant plants at 7 dpi shown in (**D**). Data represent means ± SD (*n* = 8), *** *p* < 0.001, Student’s *t*-test.

To test the role of *OsMKK6* in *Xanthomonas oryzae* pv. *oryzae* (*Xoo*) resistance, we inoculated WT and *rsr25* plants at 30 dps (days post-sowing) with *Xoo* isolate P6. At 14 days post-inoculation (dpi), leaf chlorosis was less severe in *rsr25* than in WT plants, and *rsr25* leaves had produced much shorter lesions than WT leaves (Figure 3A–C). We also tested the possible role of OsMKK6 in immunity against *Magnaporthe oryzae* (*M. oryzae*). Accordingly, we inoculated leaves of WT and *rsr25* plants at 30 dps with *M. oryzae* isolate RB22 via punch inoculation, finding that *rsr25* contained shorter lesions than the WT (Figure 3D,E). These results suggest that OsMKK6 participates in the regulation of plant immunity against both *Xoo* and *M. oryzae*.

### 2.4. OsMKK6 Regulates Pathogen-Induced Defense Responses

**Figure 4:**
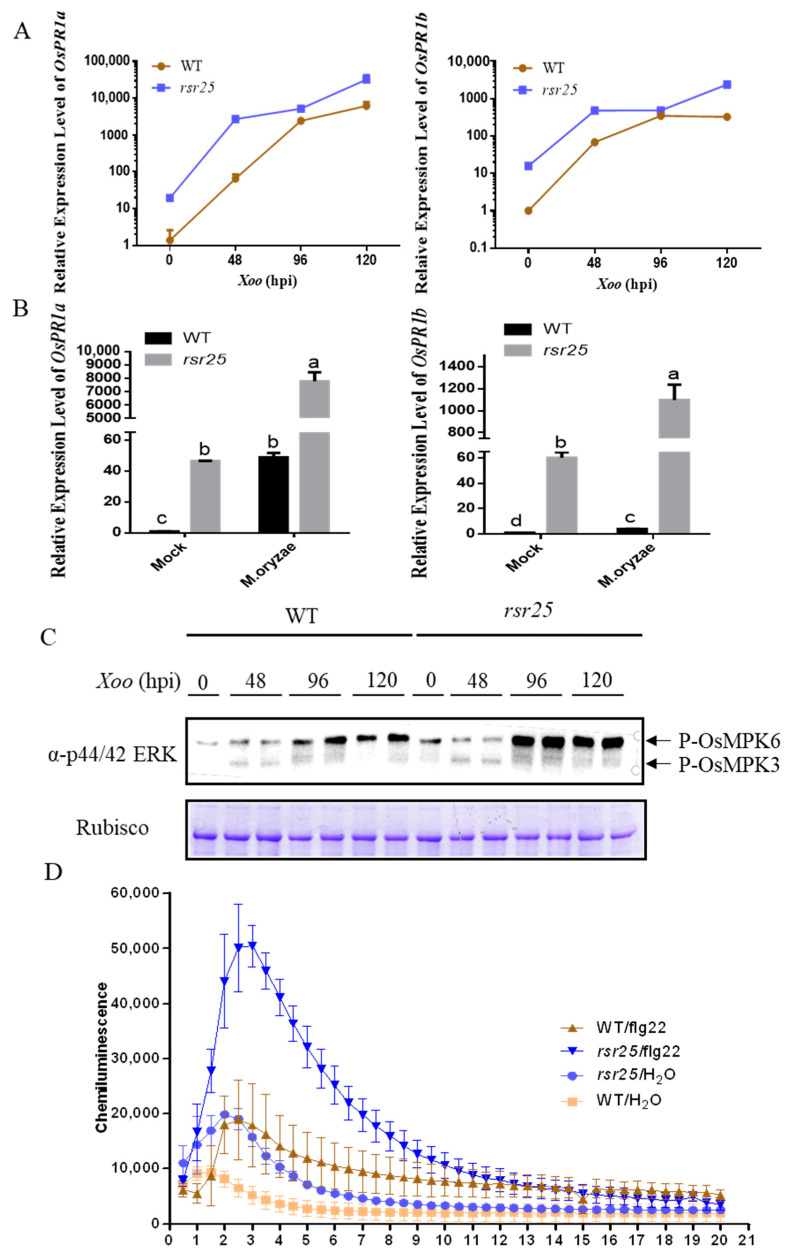
The *rsr25* mutant shows enhanced defense responses. (**A**) RT-qPCR showing *Xanthomonas oryzae* pv*.oryzae* (*Xoo*)-induced kinetic expression of *OsPR1a* and *OsPR1b* in the leaves of 3-week-old WT and *rsr25* mutant plants at the indicated time points. Data represent means ± SD (*n* = 3 independent pools of leaves, three plants per pool). (**B**) The relative expression level of *OsPR1a* and *OsPR1b* in the leaves of 3-week-old WT and *rsr25* mutant plants before *Magnaporthe oryzae* (*M. oryzae*) infection and 24 h after *M. oryzae* infection. Data represent means ± SD (*n* = 3 independent pools of leaves, three plants per pool), different letters indicate statistically significant differences at *p* < 0.05 (one-way ANOVA). (**C**) MPKs activation in WT and *rsr25* mutant plants. WT and *rsr25* mutant were inoculated with *Xoo*, and the total protein was extracted at 0 h, 48 h, 96 h, and 120 h after inoculation. MPKs activation was detected by immunoblotting with anti-p44/42 ERK antibody. CBB, coomassie blue staining. (**D**) ROS accumulation dynamics in *rsr25* mutant and WT plants after flg22 and H_2_O treatments. Data represent means ± SD (*n* = 3). hpi, hours post-inoculation.

To explore the role of OsMKK6 in plant immunity, we analyzed the expression levels of pathogenesis-related (PR) genes *PATHOGENESIS-RELATED PROTEIN 1a* (*OsPR1a*) and *OsPR1b* in WT and *rsr25* plants with or without *Xoo* and *M. oryzae* inoculation. In the absence of pathogen inoculation, the relative expression levels of *OsPR1a* and *OsPR1b* were significantly higher in *rsr25* than in WT plants (Figure 4A,B). At 48 h post-inoculation (hpi) with *Xoo*, the relative expression level of *OsPR1a* increased 40- to 80-fold in WT plants compared to uninoculated WT plants. By contrast, the relative expression level of *OsPR1a* increased 100-fold in *rsr25* compared to the untreated *rsr25* mutant. The relative expression level of *OsPR1a* continued to increase at 96 hpi and 120 hpi with *Xoo* and was significantly higher in *rsr25* than in WT plants (Figure 4A). *OsPR1b* was expressed at significantly higher levels in *rsr25* than the WT both before and after inoculation with *Xoo*, with a lower *Xoo*-induced upregulation in *rsr25* than in WT (Figure 4A), likely due to the higher basal expression level in *rsr25*. Consistent with the response to *Xoo* inoculation, *OsPR1a* and *OsPR1b* were significantly upregulated in *rsr25* compared to WT plants at 24 hpi with *M. oryzae* (Figure 4B).

OsMPK3 and OsMPK6 regulate the immune responses of rice to pathogens [18]. Therefore, we examined the phosphorylation levels of OsMPK3 and OsMPK6 in WT and *rsr25* plants before and after inoculation with *Xoo*. While there was no significant difference in the phosphorylation level of OsMPK3 before or after inoculation with *Xoo*, the phosphorylation level of OsMPK6 was significantly higher in *rsr25* than in the WT at 96 hpi and 120 hpi with *Xoo* (Figure 4C). These results indicate that the pathogen-induced immune response is stronger in *rsr25* than the WT, suggesting that OsMKK6 plays an important role in regulating the immune response in rice.

To investigate the role of OsMKK6 in PTI, we analyzed flg22-induced ROS bursts in WT and *rsr25* plants. After flg22 treatment, the peak ROS level was nearly 2.7 times higher in *rsr25* than in the WT. In the control (treated with H_2_O), the basal ROS level was 2.1 times higher in *rsr25* than in the WT (Figure 4D). These results indicate that OsMKK6 regulates flg22-induced PTI in rice. 

### 2.5. OsMKK6 Interacts with OsMPK4

**Figure 5:**
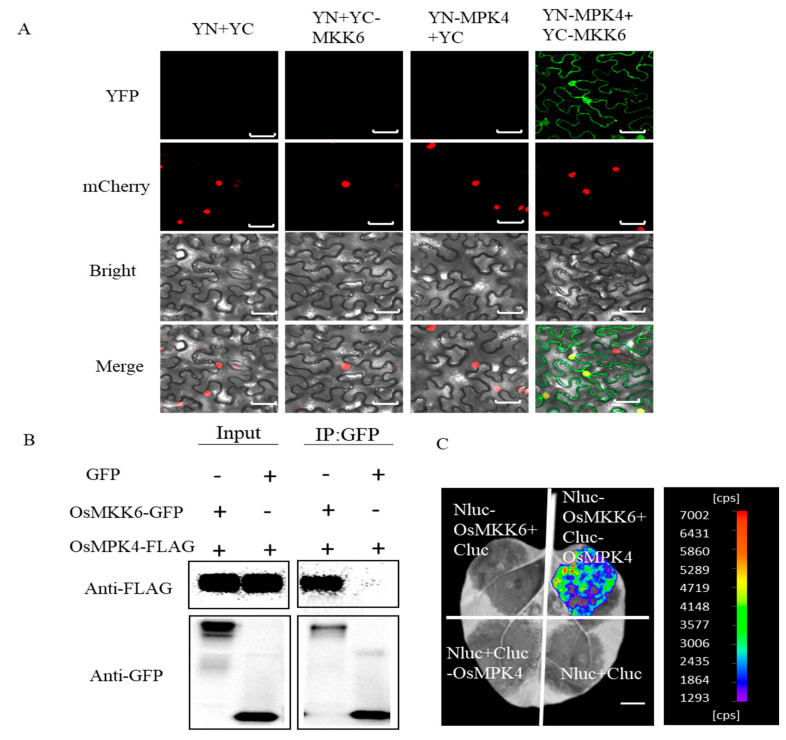
OsMKK6 interacts with OsMPK4. (**A**) BiFC assays showing the interaction between OsMKK6 and OsMPK4 in *N. benthamiana*. The OsMKK6 and OsMPK4 proteins were fused with the N- or C-terminal fragment of YFP, respectively, and transiently expressed in *N. benthamiana*, co-expressing with *H2B-mCherry* as a nuclear localization marker. YN and YC were the negative controls. The YFP signal was evaluated via confocal microscopy at 96 h after *Agrobacterium tumefaciens* infection. Scale bar = 20 μm. (**B**) The OsMKK6 interacts with OsMPK4 in Co-IP assay. GFP-tagged OsMKK6 or GFP was transiently co-expressed with FLAG-tagged OsMPK4 in *N. benthamiana.* Immunoprecipitation was carried out using anti-GFP beads. Total proteins and immunoprecipitated proteins were analyzed using anti-FLAG or anti-GFP antibodies. (**C**) OsMKK6 interacts with OsMPK4 as indicated through LCI assay. *Nluc-OsMKK6* and *CLuc-OsMPK4* were transiently expressed in *N. benthamiana* by co-infiltration; *Nluc* and *Cluc* were the negative controls. Luminescence was monitored at 48 h after *Agrobacterium tumefaciens* infection. Scale bar = 2 cm.

Arabidopsis MKK6 and MPK4 function in a MAPK cascade to prevent the autoactivation of immunity [30]. In our phylogenetic tree, OsMKK6 is in the same cluster as AtMKK6, while OsMPK4 is in the same cluster as AtMPK4 (Figure S2). To determine whether OsMKK6 and OsMPK4 function in a MAPK cascade, we tested their physical interaction by bimolecular fluorescence complementation (BiFC) assays in *Nicotiana benthamiana* leaf epidermal cells. Cells co-expressing *YN-OsMPK4* and *YC-OsMPK6* showed strong yellow fluorescent protein (YFP) signals, which overlapped with the nucleus-localized H2B-mCherry signal (Figure 5A). To further test the interaction between OsMKK6 and OsMPK4, we performed a co-immunoprecipitation (Co-IP) assay. GFP-tagged OsMKK6, but not GFP, immunoprecipitated FLAG-tagged OsMPK4 when we infiltrated the encoding constructs in *N. benthamiana* leaves (Figure 5B). We also performed firefly luciferase complementation imaging (LCI) assays in *N. benthamiana*. We detected strong luminescence in *N. benthamiana* leaves co-expressing *Nluc-OsMKK6* and *Cluc-OsMPK4* (Figure 5C). These results indicate that OsMKK6 interacts with OsMPK4 in vivo.

**Figure 6:**
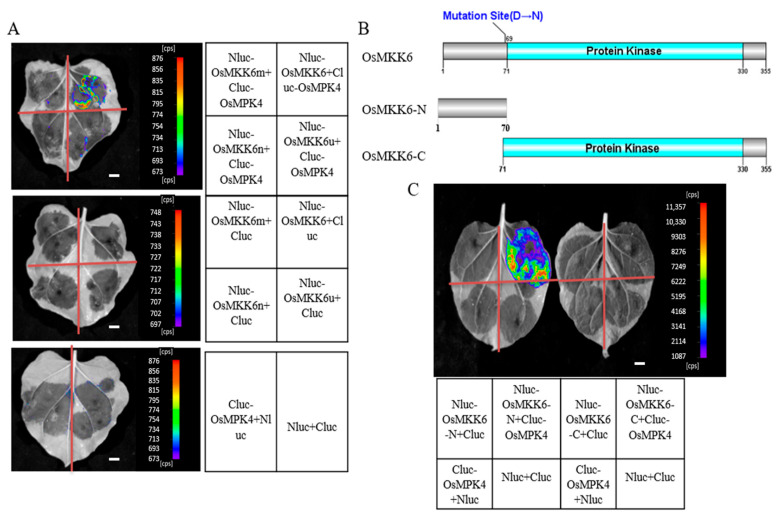
Analysis of the interaction site of OsMKK6 with OsMPK4. (**A**) The interaction of OsMKK6, OsMKK6m (69th aa, D→N), OsMKK6n (69th aa, D→Q), and OsMKK6u (69th aa, D→E) with OsMPK4 detected by LCI assay. (**B**,**C**) The interaction of OsMKK6-N and OsMKK6-C with OsMPK4 detected by LCI assay. Nluc and Cluc were the negative controls. Luminescence was monitored at 48 h after *Agrobacterium tumefaciens* infection. Scale bar = 2 cm.

To investigate whether the mutation of OsMKK6 in *rsr25* affects its interaction with OsMPK4 and whether replacing the 69th amino acid residue with other types of amino acid residues would affect the interaction of OsMKK6 with OsMPK4, we performed an LCI assay to assess the interaction between Cluc-OsMPK4 and Nluc-OsMKK6X (OsMKK6m [D69N], OsMKK6n [D69Q], and OsMKK6u [D69E]). OsMPK4 interacted only with the non-mutated form of OsMKK6, whereas OsMKK6 failed to interact with OsMPK4 after changing the D69 amino acid residue to N, Q, or E (Figure 6A). OsMKK6 contains a protein kinase domain (Figure 6B). To delineate the interaction interface between OsMKK6 and OsMPK4, we truncated OsMKK6 into OsMKK6-N (amino acids [aa] 1–70) and OsMKK6-C (aa 71–355) based on the position of its protein kinase domain (Figure 6B) and performed LCI assays to examine the interaction between Cluc-OsMPK4 and Nluc-OsMKK6-N or Nluc-OsMKK6-C. We detected a strong luminescent signal in *N. benthamiana* leaves co-expressing *Cluc-OsMPK4* and *Nluc-OsMKK6-N*, but we observed no luminescent signal in leaves co-expressing *Cluc-OsMPK4* and *Nluc-OsMKK6-C* (Figure 6C). These results suggest that OsMKK6 interacts with OsMPK4 through its N terminus (aa 1–70) and that the amino acid residue D69 plays an important role in this interaction.

### 2.6. OsMKK6 Phosphorylates OsMPK4

**Figure 7:**
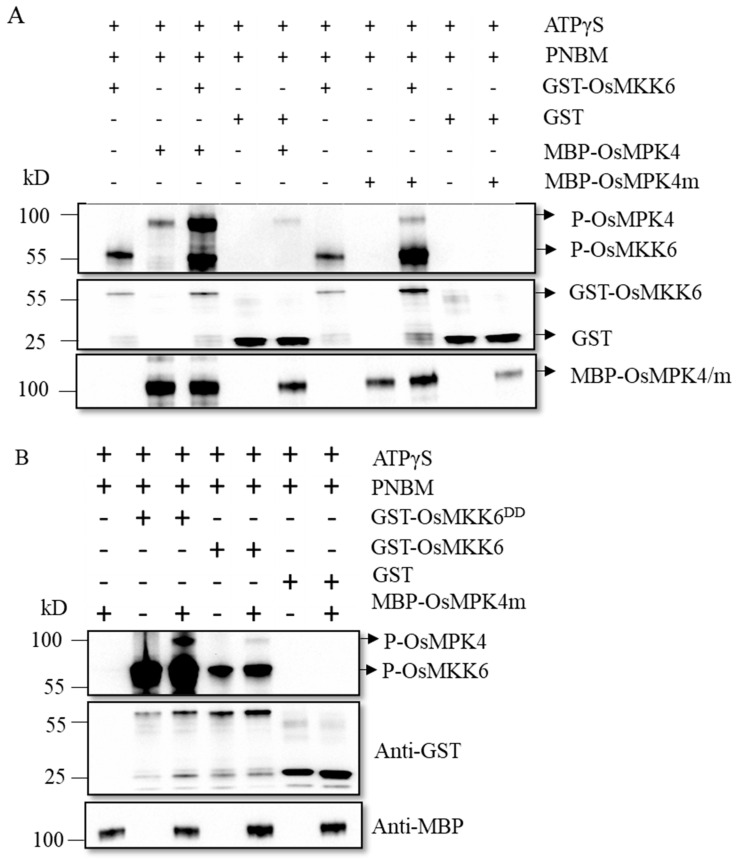
OsMKK6 phosphorylates OsMPK4 in vitro. (**A**,**B**) Phosphorylated recombinant MKK6, MKK6^DD^, MPK4, and MPK4m were detected with anti-thiophosphate ester rabbit monoclonal antibodies after gel electrophoresis (top), recombinant MKK6 and MKK6^DD^ were detected with anti-GST antibody (middle), and recombinant MPK4 and MPK4m were detected with anti-MBP antibody (bottom). Reactions lacking the specified components (− were used as controls. Recombinant proteins were separated by 10% SDS-PAGE after incubation in protein kinase buffer containing ATPγS and PNBM.

We performed in vitro phosphorylation assays using non-radioactive ATP to determine whether OsMKK6 can phosphorylate OsMPK4. We established that recombinant purified OsMKK6 and OsMPK4 are autophosphorylated in the presence of ATPγS and p-nitrobenzyl mesylate (PNBM) in vitro (Figure 7A). To avoid interference from OsMPK4 autophosphorylation, we purified MBP-OsMPK4m (harboring the D187A mutation) with a mutation in the kinase active site. MBP-OsMPK4m was not autophosphorylated, but GST-OsMKK6 phosphorylated this protein in vitro (Figure 7A). In addition, we purified a constitutively active form of OsMKK6, GST-OsMKK6^DD^ (S221D, T227D). Recombinant purified GST-OsMKK6^DD^ had a stronger ability to phosphorylate MBP-OsMPK4m compared to GST-OsMKK6 (Figure 7B). These results indicate that OsMKK6 can phosphorylate OsMPK4 in vitro.

### 2.7. The Osmpk4 Mutant Exhibits an LMM Phenotype and Enhanced Resistance to M. oryzae

**Figure 8:**
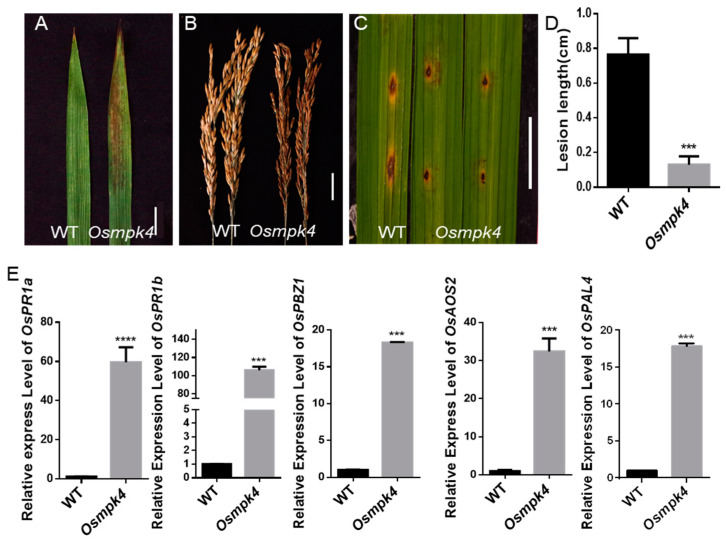
Phenotypic characterization of the *Osmpk4* mutant. (**A**) Leaves of WT and *Osmpk4* mutant plants at mature stage. Scale bar = 2 cm. (**B**) WT and *osmpk4* mutant plants panicles at mature stage. Scale bar = 2 cm. (**C**,**D**) Punch inoculation of WT and *Osmpk4* mutant plants at 60 dps (days post-sowing) with the *M. Oryzae* isolate *RB22*. (**C**) Leaves were photographed at 7 dpi (days post-inoculation). Scale bar = 2 cm. (**D**) Lesion length on the leaves of WT and *Osmpk4* mutant plants at 7 dpi shown in (**C**). Data represent means ± SD (*n* = 10), *** *p* < 0.001, Student’s *t*-test. (**E**) Comparison of the relative expression levels of defense response genes between *Osmpk4* mutant plants and WT plants at 60 dps (days post-sowing). Data represent means ± SD (*n* = 3 independent pools of leaves, three plants per pool), *** *p* < 0.001, **** *p* < 0.0001, Student’s *t*-test.

To investigate the role of OsMPK4 in the immune response of rice, we examined the phenotype of the *OsMPK4* loss-of-function mutant *Osmpk4* (R89K mutation in OsMPK4 protein) [32]. This mutant developed reddish-brown lesions on its leaves and husks, as previously reported (Figure 8A,B). Suppressing or knocking out of *OsMPK4* enhances resistance to *Xoo* in rice [35,36]. Our findings show *Osmpk4* also exhibits enhanced resistance to *M. oryzae* compared to WT (Figure 8C,D). To investigate the mechanism underlying the enhanced resistance of *Osmpk4*, we examined the transcript levels of *PR* genes in the mutant via RT-qPCR. *OsPR1a*, *OsPR1b*, *PROBENAZOLE-INDUCIBLE1* (*OsPBZ1*), *ALLENE OXIDE SYNTHASE 2* (*OsAOS2*), and *PHENYLALANINE AMMONIA-LYASE 4* (*OsPAL4*) were expressed at significantly higher levels in *rsr25* than in WT plants (Figure 8E). These results suggest that, the same as OsMKK6, OsMPK4 regulates immunity against both *Xoo* and *M. oryzae* in rice. Taken together, our findings indicate that OsMKK6 and OsMPK4 form a cascade that regulates immune responses in rice.

## 3. Discussion

Plant LMMs spontaneously produce HR-like lesions in the absence of pathogen attack; most of these mutants have improved resistance to pathogens [5]. Many LMM genes have been cloned in plants and shown to encode proteins involved in various aspects of the immune response, such as the ROS burst and signaling pathways of the phytohormones salicylic acid, jasmonate, and ethylene or components directly involved in the immune response [37]. For example, rice lesion-mimic mutant genes *SPL11* and *SDS2* are involved in the regulation of Rac1-mediated ROS bursts process [22,23]. Plants defend themselves against pathogen invasion through the PTI and ETI systems. ETI evolved in plants to detect pathogen effectors and initiate defense response. ETI is mediated by the host resistance (R) genes and recognition of pathogen effectors by R proteins can be either direct or indirect [38]. In Arabidopsis, R proteins are encoded by approximately 150 genes, and are categorized according to the structural domains they contain. R proteins generally have a very conserved protein structural domain, the leucine repeat sequence domain (LRR structural domain) [39], and many LMM are deficient for R proteins. The *suppressor of SA insensitivity 4* (*ssi4*) LMM is a gain-of-function mutant affected in a TIR-NBS-LRR factor, and constitutive activation of the SSI4 protein activates SA signaling pathways and induces the formation of chlorotic lesions [40,41]. Three R proteins of the TIR-NBS-LRR family, ACTIVATED DISEASE RESISTANCE 1 (ADR1), ADR1-LIKE 1 (ADR1-L1), and ADR1-LIKE 2 (ADR1-L2) are activators of defense responses and cell death. Loss of function of all three genes inhibits lesion formation of *lesion simulating disease1* (*lsd1*) [41,42]. NahG transgenic plants that fail to accumulate salicylic acid were crossed with multiple LMMs to determine the role of SA in immune signaling. In the presence of NahG, spontaneous lesion formation was suppressed in the lesion mimic mutants *lesion simulating disease 6* (*lsd6*), *lsd7*, *accelerated cell death 5* (*acd5*), *acd6*, *acd11*, *constitutive expressor of PR genes 22* (*cpr22*), *ssi1*, and *disease-like lesions 1* (*dll1*), suggesting that SA plays an important role in spot-like formation [43,44,45,46,47,48,49]. Therefore, LMMs are ideal materials for studying the immune responses of plants. In this study, we identified the LMM *rsr25*, which displays reddish-brown lesions on its leaves and husks (Figure 1A–C). The *rsr25* mutant exhibited poor agronomic traits, including shorter plants and panicles, and lower thousand-grain weight, seed-setting rate, and panicle number compared to WT plants (Figure 1D–H). MutMap sequencing and complementation assays identified the responsible mutated gene as being *OsMKK6* (Os01g0510100) (Figure 2). The mutation of *OsMKK6* caused the LMM phenotype of *rsr25*, suggesting that OsMKK6 might regulate the immune response in rice.

During PTI, the activation of PRRs located on the cell membrane triggers a series of immune signal transduction steps including the rapid phosphorylation of RLCKs, a burst of ROS, and activation of MAPK cascades [50]. MAPK cascades transduce immune signals to a wide range of downstream immune receptors [51]. Defects in MAPK cascade components can lead to altered immune responses in plants. In Arabidopsis, the MEKK1–MKK1/2–MPK4 cascade is activated during PTI [26,27], and defects in this cascade result in autoimmunity [28,29]. In the current study, *rsr25*, a loss-of-function mutant of *OsMKK6*, exhibited an LMM phenotype, along with increased resistance to *M. oryzae* and *Xoo* compared to the WT (Figure 3). The upregulated expression of PR genes and flg22-induced ROS production was significantly higher in *rsr25* than in WT plants in response to pathogen infection (Figure 4), suggesting that OsMKK6 might play a key role in PTI. During plant immunity, effectors secreted by pathogens can increase plant susceptibility by inhibiting PTI signaling. OsMPK4 can be dephosphorylated by SCRE6, a phosphatase effector secreted by *Ustilaginoidea virens*, which enhances OsMPK4 stability and, thus, inhibits the immune response in rice [33]. The OsMKK6–OsMPK4 cascade, therefore, plays a vital role in PTI. Whether the OsMKK6–OsMPK4 cascade interacts with other pathogen effectors should be explored to determine whether this cascade is involved in broad-spectrum disease resistance in rice.

The AtMKK6–AtMPK4 cascade negatively regulates the immune response in Arabidopsis [30]. OsMKK6 is a homolog of AtMKK6, and OsMPK4 is a homolog of AtMPK4 (Figures S1 and S2). OsMKK6 interacts with OsMPK4 in vivo and phosphorylates OsMPK4 in vitro (Figure 5 and Figure 7), suggesting that the regulation of immunity by this MAPK cascade is conserved between rice and Arabidopsis. MPKs regulate plant immunity by phosphorylating a wide range of target proteins [36]. In Arabidopsis, AtMKK6 functions simultaneously with AtMKK1 and AtMKK2 to form a MAPK cascade with AtMPK4 to prevent the activation of SUMM2 (SUPPRESSOR OF MKK1 MKK2)-mediated immunity. AtMKK6 also functions with ANP2, ANP3, and MPK4 in a separate MAPK cascade to inhibit the PHYTOALEXIN DEFICIENT 4 (PAD4)-dependent defense response, suggesting that MPK4 regulates plant immunity by targeting different substrate proteins [30]. 

Whether the OsMKK6–OsMPK4 cascade positively or negatively regulates resistance to pathogens in rice is controversial. OsMPK4 was first reported to negatively regulate resistance to *Xoo* in rice, as transgenic lines with a knockout or RNAi interference (RNAi) for *OsMPK4* exhibited enhanced resistance to *Xoo* compared to WT plants [36]. The R89K mutation of OsMPK4 improved plant resistance to *Xoo*, and transgenic lines overexpressing *OsMPK4* were more susceptible to *Xoo* than the WT [32]. Furthermore, *Ustilaginoidea virens* secretes the phosphatase SCRE6, which stabilizes OsMPK4 to suppress plant immunity [33]. These observations suggest that OsMPK4 negatively regulates rice resistance to pathogens. Notably, other studies have suggested that OsMPK4 functions in two layers of the rice–*Xoo* interaction. Both *OsMPK4* knockout and *OsMPK4*-overexpressing plants showed enhanced resistance to *Xoo* [35,36]. During the course of our study, Li et al. (2021) independently demonstrated that the OsMKK6–OsMPK4 cascade positively regulated the resistance of rice to *Xoo* by targeting OsVQ14 and OsVQ32: *OsMKK6*-RNAi plants showed no difference in resistance to *Xoo* from the WT, whereas *OsMPKK6-*overexpressing transgenic plants showed improved resistance to *Xoo* [34]. The difference in resistance to *Xoo* between *rsr25* and *OsMKK6*-RNAi transgenic plants is likely due to the presence of the D69N mutation in MKK6 in *rsr25*, which has a more severe effect on the function of OsMKK6 than the mutation employed by Li et al. (2021). We also determined that the loss of function in OsMKK6 or OsMPK4 increased the resistance of rice to *M. oryzae* (Figure 3D,E and Figure 8C,D), suggesting that the OsMKK6–OsMPK4 cascade regulates the resistance of rice to the fungal pathogen *M. oryzae*. Based on our current findings, it is not possible to determine whether OsMKK6–OsMPK4 positively or negatively regulates the immune response in rice. ENHANCED DISEASE RESISTANCE 1 (OsEDR1) negatively regulates the immune response in rice by suppressing the OsMPKK10.2–OsMPK6 cascade. The *Ossedr1* mutant shows an LMM phenotype, accumulates large amounts of H_2_O_2_ in its leaves, and shows increased resistance to *Xanthomonas oryzae* pv. *oryzicola* (*Xoc*) compared to the WT. The *Osedr1 Osmpk6* double mutant has no lesion spots on its leaves and is more susceptible to *Xoc*, suggesting that OsMPK6 positively regulates the resistance of rice to *Xoc* [21]. OsMPK6 positively regulates rice immunity by targeting OsWRKY45 [52]. In the current study, the phosphorylation level of OsMPK6 was higher in *rsr25* than in WT plants, and the *Xoo*-induced increase in OsMPK6 phosphorylation was significantly higher in *rsr25* than in WT plants at 48 h and 96 h post-inoculation (Figure 4C). These results indicate that MPK6 is activated when the OsMKK6–OsMPK4 cascade is defective. There are 8 putative MKK and 15 putative MPK genes in the rice genome [38], suggesting that MKKs likely activate multiple MPKs; the crosstalk between various immune signaling pathways in rice might occur at this level. After receiving external signals, plant MAPKKKs mostly phosphorylate the two conserved serine (S) and threonine (T) residues in the S/T-X5-S/T (X is any amino acid) motif of MKKs and activate MKKs. The activated MKKs phosphorylate both the threonine (T) and the tyrosine (Y) in the T-D-Y or T-E-Y motif of MPKs and activate MPKs [53]. The genetic compensation response, which was first reported in zebrafish (*Danio rerio*), describes how the knockout of one gene has no phenotypic effect due to genetic compensation by other homologous genes whose expression is stimulated [54,55]. We propose that, if the OsMKK6–OsMPK4 cascade is defective, an enhanced genetic compensation response may occur in rice as well, causing another cascade to be activated. Whether the formation of lesion spots in the *rsr25* mutant results from the activation of OsMPK6 requires further study, such as examining whether the loss of function of *OsMPK6* in the *rsr25* mutant can suppress its phenotype. The next steps are to verify whether OsMPK6 is also activated in *osmpk4* mutant by in vivo phosphorylation assay and to develop *Osmkk6 Osmpk6* and *Osmpk4 Osmpk6* double mutant materials to further investigate the relationship between the OsMKK6–OsMPK4 cascade and OsMPK6 in regulating the immune response of rice through phenotypic observation. In conclusion, we examined the phenotype of an *OsMKK6* loss-of-function mutant. We demonstrated that OsMKK6 plays a vital role in the resistance of rice to pathogens. We also demonstrated that the OsMKK6–OsMPK4 cascade is involved in regulating the resistance of rice to the fungal pathogen *M. oryzae*, providing new clues for further studying the roles of MAPK cascades in rice immunity.

## 4. Materials and Methods

### 4.1. Plant Materials and Growth Conditions

The *rsr25* mutant was isolated from an ethyl methanesulfonate (EMS) mutant library of the rice (*Oryza sativa*) cultivar Xiushui134 (XS134, wild type [WT]). The *rsr25* mutant was crossed with XS134 to obtain an F_1_ population. The F_2_ population was obtained by selfing F_1_ plants and was subjected to MutMap sequencing. The *Osmpk4* mutant and its corresponding wild type (Chang Geng 3, CG3) were a gift from the China National Rice Research Institute; the loss-of-function *Osmpk4* mutant was described previously [29]. Plants from the F_2_ population were grown in Hainan, China (N:18°, E:109°). The agronomic traits of wild type and *rsr25* plants were measured in Changxing (N:30°, E:119°). Plants used for phenotypic analysis and molecular biology experiments were grown in a growth chamber at 30 °C/25 °C (day/night) and 60% to 70% relative humidity, with light at a photon density of 300 µmol m^−2^ s^−1^ supplied by light bulbs and a photoperiod of 12 h light/12 h dark. The plants were grown in Kimura nutrient solution [56], which was changed once per week. 

### 4.2. Vector Construction

To construct the complementation vector *proOsMKK6:OsMKK6CDS-GFP*, the promoter sequence of *OsMKK6* (3000 bp upstream of ATG) was amplified from XS134 genome DNA, and the CDS (Coding sequence) of *OsMKK6* (with the stop codon removed) was amplified from the XS134 cDNA. Those two sequences were amplified together using overlapping extension PCR and ligated into the pCAMBIAI1300-GFP vector. The *proOsMKK6:OsMKK6CDS-GFP* vector was introduced into *rsr25* mutant by *Agrobacterium tumefaciens*-mediated transformation.

To construct the *35S:OsMKK6-GFP* vector, the CDS of *OsMKK6* was ligated into the pCAMBIAI1300-35S-GFP vector.

To construct the *GST-OsMKK6* vector, the CDS of *OsMKK6* was ligated into the pGEX-4T-1 vector. The point mutation in OsMKK6 (the 221st serine residue and 227th threonine residue were replaced with aspartate residue) was introduced using an overlap extension PCR, and then the mutated *OsMKK6* CDS sequence was ligated into pGEX-4T-1 vector to generate *GST-OsMKK6^DD^* vector. To generate *MBP-OsMPK4* and *MBP-OsMPK4m* vectors, the CDS of *OsMPK4* and mutated *OsMPK4* CDS sequence were ligated into the MBP-pET-28a vector. The point mutation in *OsMPK4* (the 187th aspartate residue was replaced with alanine residue) was introduced using an overlap extension PCR. The CDS of *OsMPK4* was ligated into the PTCK303-FLAG vector to obtain the *Ubi:OsMPK4-FLAG* vector.

The CDS of *OsMKK6* was ligated into the p35Spro-YFP^C^ vector to obtain the *YC-OsMKK6* vector. To generate the *YN-OsMPK4* construct, the CDS of *OsMPK4* was ligated into the p35Spro-YFP^N^ vector.

To construct the *Cluc-OsMPK4* vector, the CDS of *OsMPK4* was ligated into the Cluc vector. To generate *Nluc-OsMKK6*, *Nluc-OsMKK6m* (the 69th aspartic was replaced with asparagine residue), *Nluc-OsMKK6n* (the 69th aspartic was replaced with glutamine residues), *Nluc-OsMKK6u* (the 69th aspartic was replaced with glutamic acid residues), *Nluc-OsMKK6-N* (1-70 amino acid residues of OsMKK6), and *Nluc-OsMKK6-C* (71-355 amino acid residues of OsMKK6) vectors, the CDS of *OsMKK6* and mutated *OsMKK6* CDS sequences were ligated into the Nluc vector. The primers for vectors are listed in Table S2.

### 4.3. In Vitro Phosphorylation Assays 

In vitro phosphorylation assays were performed as previously described [57]. Recombinant glutathione S-transferase (GST)-OsMKK6 or GST-OsMKK6^DD^ (500 ng) was incubated with the substrate proteins maltose binding protein (MBP)-OsMPK4 or MBP-OsMPK4m (1 μg) in kinase reaction buffer (30 mM Tris-HCl pH 7.5, 1 mM EGTA, 10 mM MgCl_2_, 1 mM DTT, and 3 μL 10 mM ATPγS [Abcam, ab138911]) at 30 °C for 30 min. After adding 1.5 μL 50 mM p-Nitrobenzyl mesylate (PNBM, Abcam, Cambridge, UK; ab138910) to the reactions, the samples were incubated at 30 °C for 1.5 h. Recombinant proteins were separated by 10% (*w*/*v*) SDS-PAGE, and phosphorylated recombinant MKK6, MKK6^DD^, MPK4, and MPK4m were detected by immunoblotting with anti-thiophosphate ester rabbit monoclonal antibodies (Abcam, Cambridge, UK; ab92570, 1:5000).

To detect MAPK activation, WT and *rsr25* plants were inoculated with *Xoo*, and total proteins were extracted from the plants at 0 h, 48 h, 96 h, and 120 h after inoculation. MPK activation was detected by immunoblotting with an anti-p44/42 ERK antibody (Cell Signaling, Danvers, MA, USA; 4370S).

### 4.4. Pathogen Infection

Detached leaves from one-month-old plants were subjected to punch inoculation with *M. oryzae.* Then, 5 μL of a spore suspension of *M. oryzae* isolate RB22 (5 × 10^5^ spores/mL in 0.05% [*v*/*v*] Tween-20) was added to holes in the leaves that were created by gentle punching with a pipette tip. Following inoculation, the leaves were incubated at 25 °C for 24 h in the dark and switched to a 12 h light/12 h dark photoperiod at 25 °C. Lesion length was measured at seven days post-inoculation (dpi). 

Three-week-old seedlings were sprayed with a spore suspension of *M. oryzae* isolate RB22 (5 × 10^5^ spores/mL in 0.05% [*v*/*v*] Tween-20) or 0.05% (*v*/*v*) Tween-20 as a control. The inoculated seedlings were grown in a growth chamber under the same growth conditions, except that the relative humidity was adjusted to 95%. Total RNA was extracted from the seedlings at 24 h post-inoculation (hpi).

For the *Xoo* incubation assay, the concentration of *Xoo* strain P6 in the liquid medium (20 g/L sucrose; 5 g/L peptone; 0.25 g/L MgSO_4_-7H_2_O; 0.5 g/L K_2_HPO_4_; pH 7.2~7.5) was adjusted to 10^9^ cells/mL (OD_600_ = 1.0). For inoculation, scissors were dipped into the bacterial suspension and used to remove leaf tips (5 cm). The inoculated plants were grown in a growth chamber under the same conditions described above, except that the relative humidity was adjusted to 95%. Lesion length was measured at 14 dpi. 

### 4.5. Measurement of ROS

ROS was measured in flg22-treated seedlings as previously described [17]. Briefly, leaf discs were collected from the leaves of 3-week-old wild type and *rsr25* seedlings using a 1.5 mm diameter Miltex puncher. The leaf discs were immersed in ddH_2_O for 12 h to eliminate the effects of physical damage. Two leaf discs per sample were placed into the bottom of a 1.5 mL centrifuge tube with 100 μL luminol (Bio-Rad Immun-Star Horseradish Peroxidase Substrate, Hercules, CA, USA, 170-5040), 1 μL horseradish peroxidase, and 100 nM flg22 or ddH_2_O as a control. Luminescence was measured immediately in a Glomax 20/20 photometer (Promega, Madison, WI, USA) at 30 s intervals over a 20 min period, with each seedling subjected to three biological replicates.

### 4.6. Bimolecular Fluorescence Complementation (BiFC) Assays

The coding sequence of *OsMKK6* was ligated into the p35Spro-YFP^C^ vector, and the coding sequence of *OsMPK4* was ligated into the p35Spro-YFP^N^ vector. The constructs were transiently expressed in *Nicotiana benthamiana* leaf epidermal cells by Agrobacterium (*Agrobacterium tumefaciens*)-mediated infiltration and co-expressed with *H2B-mCherry* (encoding a fusion between histone H2B and mCherry) as a nuclear localization marker. p35Spro-YFP^C^ (YC) and p35Spro-YFP^N^ (YN) were used as negative controls. The YFP and mCherry signals were imaged under a Zeiss 595 LSM710NLO confocal laser (Oberkohen, Battenwürburg, Germany) scanning microscope at 96 h after infiltration.

### 4.7. Luciferase Complementation (LCI) Assays

The constructs *Nluc-OsMKK6*, *Nluc-OsMKK6m*, *Nluc-OsMKK6n*, *Nluc-OsMKK6u*, *Nluc-OsMKK6-N*, *Nluc-OsMKK6-C*, and *Cluc-OsMPK4* were transiently expressed in *N. benthamiana* by Agrobacterium-mediated infiltration; Nluc and Cluc were used as the negative controls. The *N. benthamiana* leaves were sprayed with luciferase substrate (150 μg/mL D-Luciferin potassium salt) at 48 h after infiltration. Luminescence was monitored 5 min later using a BERTHOLD LB985 imaging camera (Bad Wildbad, Schwartzwald, Germany).

### 4.8. Co-IP Assay

Constructs encoding GFP-tagged OsMKK6 or GFP alone were transiently co-expressed with *OsMPK4-FLAG* in *N. benthamiana* leaves. Total proteins were extracted from the samples with lysis buffer (50 mM Tris-HCl pH 7.4, 150 mM NaCl, 1 mM EDTA, 10% [*v*/*v*] glycerol, 1% [*v*/*v*] Triton X-100, 1 mM PMSF, 20 mM MG132, and one tablet protease cocktail per 15 mL), and the immunoprecipitated proteins were incubated with anti-GFP magnetic beads (ABclonal, WuHan, China AE079). The immunoprecipitated proteins were separated by SDS-PAGE (12% gel) and analyzed by immunoblotting using anti-FLAG (Sigma-Aldrich, St. Louis, MO, USA, F1804) or anti-GFP antibodies (Sigma-Aldrich, USA; SAB4701015). Following incubation with the corresponding secondary antibody (anti-mouse IgG HRP-linked antibody [CST, Danvers, MA, USA; 7076V] and goat anti-mouse HRP antibody [Licor, Lincoln, Nebraska, USA; 926-80011]) for 1 h, the immunoblot signal was visualized using Immobilon Western HRP substrate (Merck Millipore, Darmstadt, Schwartzwald, Germany; WBKLS0100).

### 4.9. RNA Extraction and RT-qPCR

Total RNA was extracted from WT, *rsr25*, CG3, and *Osmpk4* leaves using a TaKaRa MiniBEST Plant RNA Extraction Kit (TaKaRa, Kusatsu, Shiga, Japan; 9769S). First-strand complementary DNA (cDNA) was obtained using a PrimeScript^™^RT reagent Kit with gDNA Eraser (TaKaRa, RR047A). qPCR analysis was performed using TB Green Fast qPCR Mix (TaKaRa, RR430s) in a LightCycler 480 Real-Time PCR System (Roche, Basel, Switzerland). The 2^−ΔΔ^CT method was used to analyze the RT-qPCR data, and the housekeeping gene *OsACTIN* (LOC_Os03g50885) was used as an internal control. The primers used for RT-qPCR analysis are listed in Table S3.

### 4.10. Subcellular Localization Assay

The construct-encoding OsMKK6-GFP or GFP (control) was co-transfected with *mCherry* in rice protoplasts or transiently co-expressed with *H2B-mCherry* in *N. benthamiana*. The GFP and mCherry signals were detected under an LSM710 confocal laser scanning microscope (Zeiss, Oberkohen, Battenwürburg, Germany).

### 4.11. Analysis of Protein Sequences and Phylogenetic Analysis

The relevant protein sequences were downloaded from NCBI or obtained by sequencing and placed in a txt file in fixed format. The protein sequences were compared using ClustalX1.83 software. The evolutionary tree was drawn with MEGA software (MEGA5.0) using the minimal evolution method with 1000 bootstrap replicates [58]. The protein names and loci encoding MKKs and MPKs were described previously [31].

### 4.12. Accession Numbers

The sequences in the article can be downloaded from the Rice Annotation Project (RAP, https://rapdb.dna.affrc.go.jp/index.html accessed on 21 June 2022), and the corresponding locus numbers are as follows: OsMKK6, Os01g0510100; OsMPK4, Os10g0533600; OsPBZ1, Os12g0555500; OsPR1a, Os07g0129200; OsAOS2, Os03g0225900; OsPR1b, Os01g0382000; OsPAL4, Os02g0627100. The protein names and gene loci of MKKs and MPKs in the paper refer to article [31].

## Data Availability

Not applicable.

## Supplementary Materials

The supporting information can be downloaded at: https://www.mdpi.com/article/10.3390/ijms241612678/s1.
