# Supplementary material for: OsMKK6 Regulates Disease Resistance in Rice
Source: Int J Mol Sci. 2023 Aug 11;24(16):12678. doi: 10.3390/ijms241612678 (PMC10454111; doi:10.3390/ijms241612678)
Supplement: Supplementary file 1 [file ijms-24-12678-s001.zip › ijms-2523509-SI.pdf]

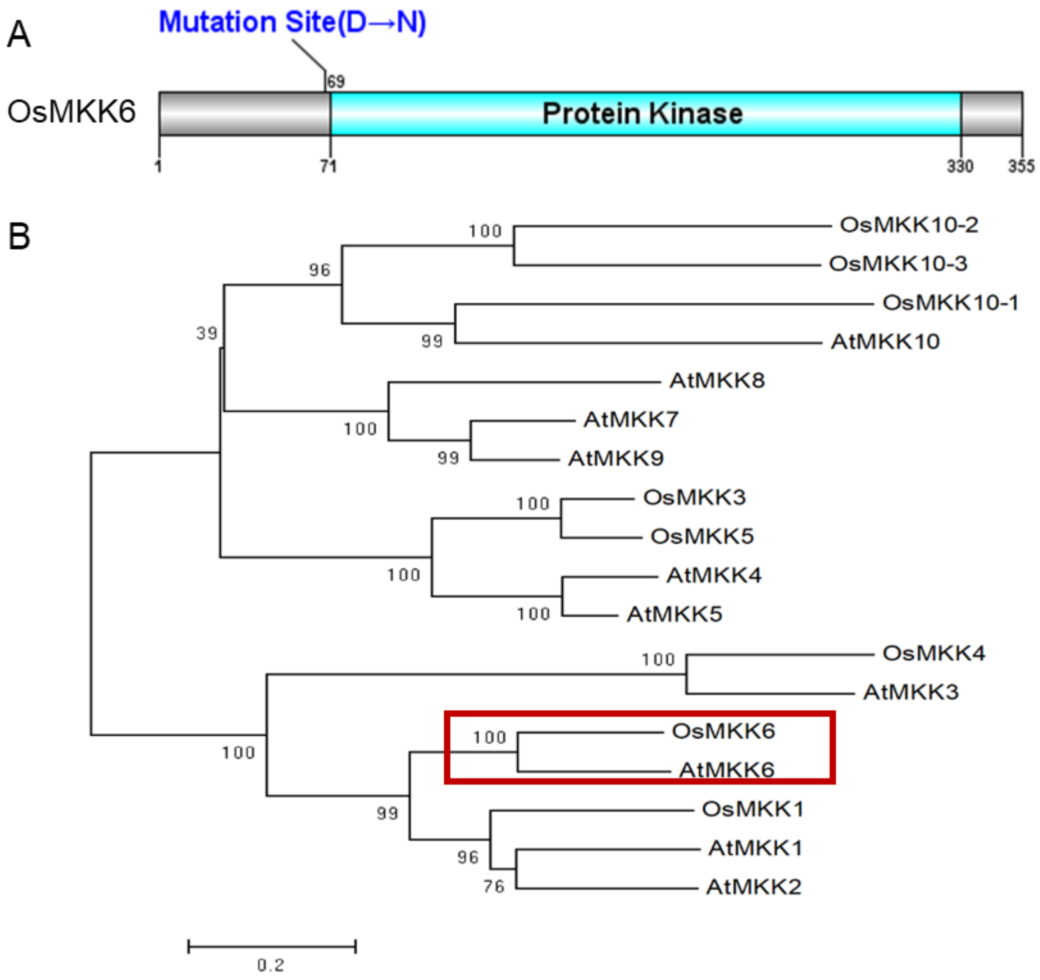

Figure S1. Conserved domains of OsRSR25 protein and phylogenetic tree of MKKs in rice and *Arabidopsis thaliana.* **(A)** Conserved domains of OsRSR25 protein, the numbers in the figure indicate the location of amino acid residues. **(B)** Phylogenetic tree of MKKs in rice and *Arabidopsis thaliana*, the red box indicates the phylogenetic branch of OsRSR25 and its homologous proteins in *Arabidopsis*. Os: *Oryza sativa*, At: *Arabidopsis thaliana*. The protein names of MKKs in the figure refer to the article(Hamel et al., 2006).

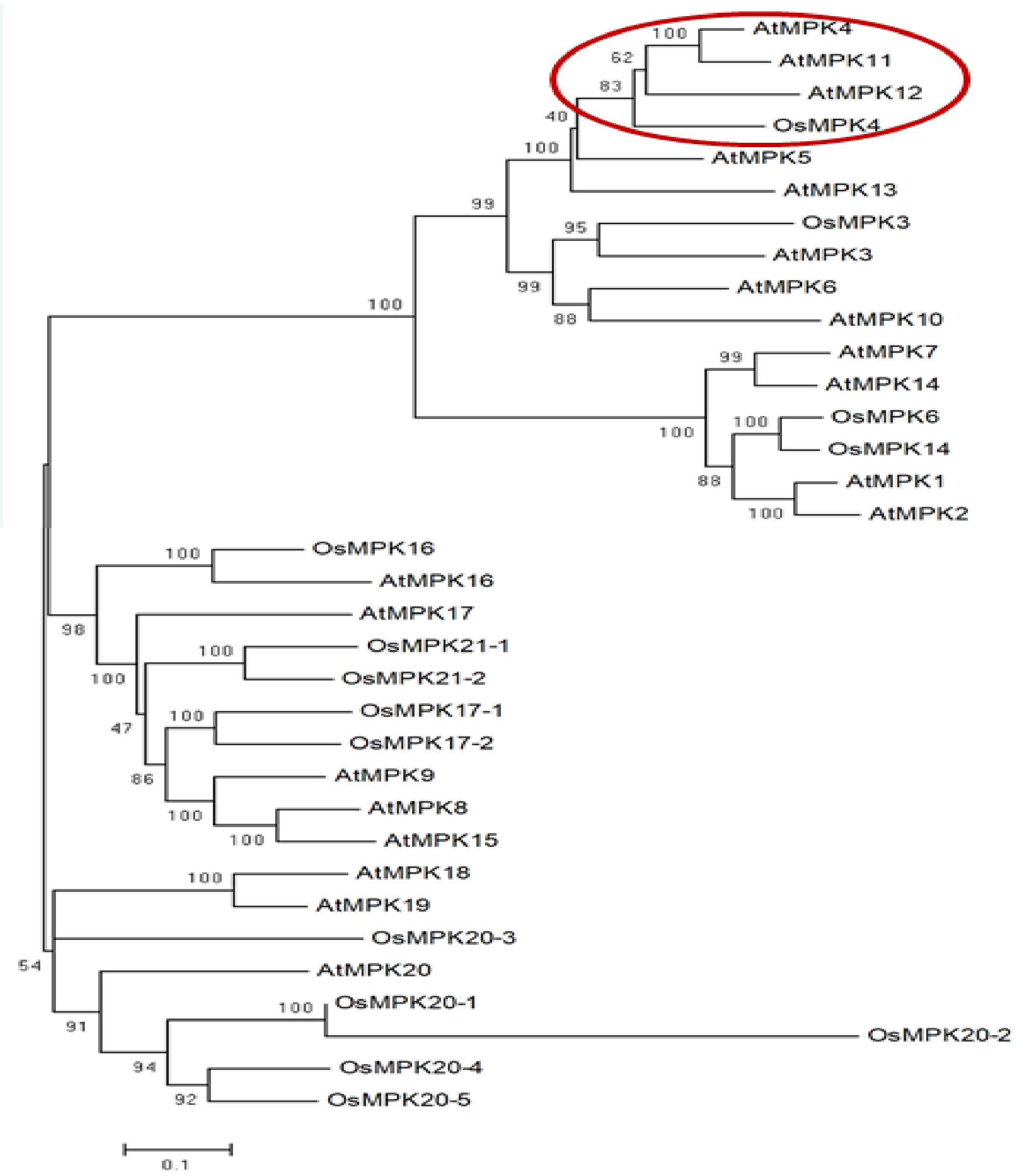

Figure S2. Phylogenetic tree of MPKs in rice and *Arabidopsis thaliana.*

Generated in MEGA5, the red circle indicates the phylogenetic branch of OsMPK4 and its homologous proteins in *Arabidopsis*. Os: *Oryza sativa*, At: *Arabidopsis thaliana*, The protein names and gene locus of MPKs in the figure refer to the article(Hamel et al., 2006).

Table S1. F_2 generation segregation ratio after crossing the *rsr25* mutant with XS134.

	Actual number	Theoretical number	**χ2**
Normal phenotype	245	240	
Lesion mimic phenotype	75	80	

Table S2. Primers for the vectors.

Primer name	Sequence（5' to 3'）
35S:OsMKK6-GFP-F	TCCGAATTCGAGCTCATGAGGGGGAAGAAGCCGCA
35S:OsMKK6-GFP-R	GTGGTGGTGCTCGAGCTCGGATATATTCATTGGA
Ubi:OsMPK4-FLAG-F	GGATCCCCGGGTACCATGGATTCCTCCTCCGGCGG
Ubi:OsMPK4-FLAG-R	GTAGTCCATACTAGTGTAGGGAGGATCCGGATTAA
GST-OsMKK6-F	GGATCCCCGGAATTCATGAGGGGGAAGAAGCCGCA
GST-OsMKK6-R	GCCGCTCGAGTCGACTTACTCGGATATATTCATTG
MKK6m-overlap-F	AGATGTTCAGTTGTCAATGAATGACCTTGAGATGATTCA
MKK6m-overlap-R	TGAATCATCTCAAGGTCATTCATTGACAACTGAACATCT
MKK6n-overlap-F	TTCAGTTGTCAATGGAAGACCTTGAGAT
MKK6n-overlap-R	ATCTCAAGGTCTTCCATTGACAACTGAA
MKK6u-overlap-F	TTCAGTTGTCAATGCAAGACCTTGAGAT
MKK6u-overlap-R	ATCTCAAGGTCTTGCATTGACAACTGAA
MKK6DD-overlap-F	CCAACAAAGTCATCCCGCTGACCCATGTCACTTGCTAGCA
MKK6DD-overlap-R	TGCTAGCAAGTGACATGGGTCAGCGGGATGACTTTGTTGG
Nluc-MKK6-F	GACGAGCTCGGTACCATGAGGGGGAAGAAGCCGCA
Nluc-MKK6-R	CGAGATCTGGTCGACCTCGGATATATTCATTGGA
Nluc-MKK6-N-F	GACGAGCTCGGTACCATGAGGGGGAAGAAGCCGCA
Nluc-MKK6-N-R	CGAGATCTGGTCGACGTCATCCATTGACAACTGAA
Nluc-MKK6-C-F	GACGAGCTCGGTACCATGCTTGAGATGATTCAAGTCAT
Nluc-MKK6-C-R	CGAGATCTGGTCGACCTCGGATATATTCATTGGA
Cluc-OsMPK4-F	TCCCGGGGCGGTACCATGGATTCCTCCTCCGGCG
Cluc-OsMPK4-R	ACGAAAGCTCTGCAGTTAGTAGGGAGGATCCGGA
MBP-OsMPK4-F	TCCGAATTCGAGCTCATGGATTCCTCCTCCGGCGG
MBP-OsMPK4-R	GTGGTGGTGCTCGAGGTAGGGAGGATCCGGATTAA
OsMPK4m-overlap-F	GATCTCAAGATTGCTGCCTTTGGGCTTGCAAGA
OsMPK4m-overlap-R	TCTTGCAAGCCCAAAGGCAGCAATCTTGAGATC
YN-OsMKK6-F	TTACGAACGATAGTTAATTAACATGAGGGGGAAGAAGCCG

YN-OsMKK6-R	CCTCCTCCACTAGTGGCGCGCCCCTCGGATATATTCATTGGA
YC-OsMPK4-F	TTACGAACGATAGTTAATTAACATGGATTCCTCCTCCGGCG
YC-OsMPK4-R	CCTCCTCCACTAGTGGCGCGCCCGTAGGGAGGATCCGGA

Table S3. Primers used for qRT-PCR.

Primer name	Sequence（5’ to 3’）
QPCR-PAL4-F	GTCAACTCTCTTGGACTCATCT
QPCR-PAL4-R	GATCAAGAACGTGGAGGACAT
OsPBZ1-RT-qPCR-F	TCTACACCATGAAGCTTAACCC
OsPBZ1-RT-qPCR-R	CATCCGACTTTAGGACATGACT
OsPR1b-RT-qPCR-F	CGTCTTCATCACATGCAACTAT
OsPR1b-RT-qPCR-R	ACGCCCGTGTGTATAAATAACT
OsPR1a-RT-qPCR-F	GAAGTACGGCGAGAACATCTTC
OsPR1a-RT-qPCR-R	GTACCACTGCTTCTCCGAC
OsAOS2-RT-qPCR-F	CGATCAAGAAGGGGGAGATG
OsAOS2-RT-qPCR-R	CATTCGACCAGTACACGTATTG
Actin-F	CAACACCCCTGCTATGTACG
Actin -R	CATCACCAGAGTCCAACACAA
